# Interface Coordination Engineering of P-Fe_3_O_4_/Fe@C Derived from an Iron-Based Metal Organic Framework for pH-Universal Water Splitting

**DOI:** 10.3390/nano13131909

**Published:** 2023-06-22

**Authors:** Minmin Fan, Peixiao Li, Baibai Liu, Yun Gong, Chengling Luo, Kun Yang, Xinjuan Liu, Jinchen Fan, Yuhua Xue

**Affiliations:** 1 School of Materials and Chemistry, University of Shanghai for Science and Technology, Shanghai 200093, China; fanminmin1998@163.com (M.F.); 211590181@st.usst.edu.cn (Y.G.); clingluo@163.com (C.L.); yangkun199800@163.com (K.Y.); jcfan@usst.edu.cn (J.F.); xueyuhua@usst.edu.cn (Y.X.); 2 Beijing Smartchip Microelectronics Technology Company Limited, Beijing 102200, China; pricheer@126.com; 3 Key Laboratory of Optoelectronic Technology & Systems (Ministry of Education), College of Optoelectronic Engineering, Chongqing University, Chongqing 400044, China; 15067132581@163.com

**Keywords:** interface coordination engineering, donor-like catalyst, under-coordinated atoms, water splitting

## Abstract

Developing electrocatalysts with high energy conversion efficiency is urgently needed. In this work, P-Fe_3_O_4_/Fe@C electrodes with rich under-coordinated Fe atom interfaces are constructed for efficient pH-universal water splitting. The introduction of under-coordinated Fe atoms into the P-Fe_3_O_4_/Fe@C interface can increase the local charge density and polarize the 3d orbital lone electrons, which promotes water adsorption and activation to release more H^*^, thus elevating electrocatalytic activity. As a donor-like catalyst, P-Fe_3_O_4_/Fe@C displays excellent electrocatalytic performance with overpotentials of 160 mV and 214 mV in acidic and alkaline electrolytes at 10 mA cm^−2^, in addition to pH-universal long-term stability.

## 1. Introduction 

Electrochemical water-splitting to produce high purity hydrogen is an effective solution owing to the low cost, high purity, and eco-friendliness. A high overpotential is necessary to impel the hydrogen evolution reaction (HER). To date, significant endeavors have been devoted to explore high-performance HER electrocatalysts with a low overpotential, including sulphide, carbide, nitride, phosphide, and so on [1,2,3,4,5]. However, they are limited due to inferior electrical conductivity, leading to sluggish reaction kinetics, low energy conversion efficiency, and poor cycling stability [6,7,8]. Therefore, developing HER electrocatalysts with high energy conversion efficiency is urgently needed for pH-universal water-splitting.

Recently, iron-based materials have received intense attention because of their relatively lower intrinsic electrical resistivity, abundant reserves, and inexpensive metallic element; therefore, iron has been widely studied as an electrocatalyst [9]. Iron phosphides show excellent electrocatalytic HER activity due to highly active hydrogenases [10,11]. However, it suffers from poor operation stability owing to the high oxygen sensitivity of iron phosphide [12]. Fortunately, Fe_3_O_4_ exhibits superb electrical conductivity compared with other metal oxides because of the electron transfer between Fe (III) and Fe (II) [13,14,15]. In addition, Fe_3_O_4_ shows a low water dissociation energy to rapidly break the H-O bond [16,17,18]. Based on the above advantages, Fe_3_O_4_ has shown promising potential as an electrocatalyst [19,20,21,22]. It has been reported that iron-based oxide/hydroxides show good OER activity, yet their application for HER is relatively rare.

The interface characterization of electrocatalysts is all-important for HER. Constructing a rich interface based on Fe_3_O_4_ is considered to be a relatively effective means, which can not only adjust the electronic structure in the upper edge of the valence band but also provide more active sites and expedite charge transfer, thus enhancing the HER activity. Owing to the bond order–length–strength (BOLS) correlation and nonbonding electron polarization (NEP) effect [23], Fe nanostructures with rich under-coordinated atoms can fascinate more electrons to facilitate the Volmer step in the HER process. Therefore, it is an attractive means to construct P-Fe_3_O_4_/Fe@C with rich under-coordinated atom interfaces for efficient pH-universal HER. Herein, we report an interface coordination engineering of P-Fe_3_O_4_/Fe@C by introducing proper under-coordinated Fe atom ratios into interfaces for acidic and alkaline HER. MIL-53(Fe) has been chosen as the precursor to synthesize the P-Fe_3_O_4_/Fe@C. By adjusting the pyrolysis temperature, the under-coordinated Fe atom ratios in the P-Fe_3_O_4_/Fe@C interfaces can be effectively controlled. It is expected that the introduction of proper under-coordinated atom ratios in the P-Fe_3_O_4_/Fe@C interfaces will significantly elevate their HER activity across a wide pH range. However, little attention has been paid to study the influence of interface characterization on the HER performance of P-Fe_3_O_4_/Fe@C. The electrocatalytic mechanism was also studied, except for the characterization of HER activity for P-Fe_3_O_4_/Fe@C.

## 2. Experimental 

### 2.1. Synthesis of MIL-53(Fe)

Briefly, 540 mg FeCl_3_·6H_2_O and 344 mg 1,4-benzenedicarboxylate (H_2_BDC) were dispersed into N,N-dimethylformamide (40.88 g, DMF) under magnetic stirring for 30 min. Then, the above solution was transferred to the autoclave and reacted at 150 °C for 15 h. After the reaction, the obtained precipitate (MIL-53(Fe)) was filtrated, washed three times with DMF and methanol, and dried at 80 °C for 12 h.

### 2.2. Fabrication of P-Fe_3_O_4_/Fe@C Catalysts

Firstly, MIL-53(Fe) powder was carbonized at 600, 700, 800, and 900 °C for 2 h at a heating rate of 2 °C min^−1^ in an Ar atmosphere. After carbonation, MIL-53(Fe) was transformed into the Fe_3_O_4_/Fe@C hybrids. By adjusting the pyrolysis temperature, the ratios of Fe_3_O_4_ and Fe can be effectively controlled. Then, 0.1 g Fe_3_O_4_/Fe@C hybrids and 0.7 g NaH_2_PO_2_·H_2_O were placed into two different porcelain boats in a sealed crucible and then heated to 300 °C for 2 h. The final high purity products, which are P-Fe_3_O_4_/Fe@C hybrids, were washed three times with ethanol and DI water, and dried at 70 °C for 24 h. The P-Fe_3_O_4_/Fe@C hybrids obtained at 600, 700, 800, and 900 °C were named as FCP600, FCP700, FCP800, and FCP900, respectively.

## 3. Results and Discussion

### 3.1. Characterization

**Figure 1:**
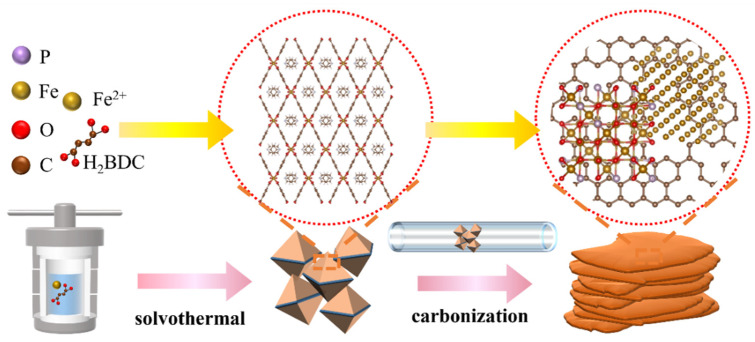
Synthesis process diagram of P-Fe_3_O_4_/Fe@C hybrids.

Figure 1 shows the synthetic procedure of P-Fe_3_O_4_/Fe@C hybrids. MIL-53(Fe) is constructed by choosing H_2_BDC as the organic building block related to Fe^3+^ as the inorganic metal center. MIL-53(Fe) is connected by an FeO_4_(OH)_2_ octahedron and H_2_BDC. After the thermal treatment in an Ar atmosphere, the Fe^3+^ in the MIL-53(Fe) is transformed into the Fe_3_O_4_ with a certain amount of metal Fe. At the same time, the organic building blocks are pyrolyzed to amorphous carbon to form the Fe_3_O_4_/Fe@C hybrids. After the in-situ pyrolysis process in the presence of sodium hypophosphite, the sodium hypophosphite is decomposed to form the PH_3_ in the Ar atmosphere at 300 °C. The P atoms are doped by the reaction of PH_3_ and Fe_3_O_4_/Fe@C hybrids to form the P-Fe_3_O_4_/Fe@C hybrids.

**Figure 2:**
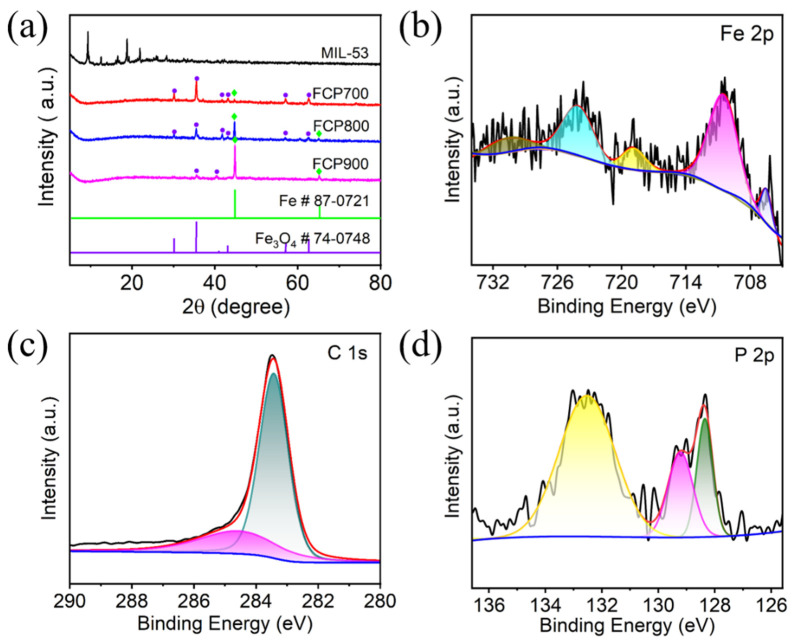
(**a**) XRD patterns of MIL-53(Fe), FCP700, FCP800, and FCP900; high-resolution XPS spectra of (**b**) Fe 2p, (**c**) C 1 s, and (**d**) P 2p of FCP800.

The XRD results in Figure 2a demonstrate the phase and crystal structure of MIL-53(Fe), FCP700, FCP800, and FCP900. The XRD pattern of MIL-53(Fe) is similar to the previously reported result (CIF: 690314-690316) [24,25], demonstrating that the MIL-53(Fe) is successfully prepared. MIL-53(Fe) shows strong diffraction peaks, indicating good crystallinity. The XRD pattern of FCP600 has been provided in Figure S1. The main diffraction peaks at 24.1°, 33.2°, 35.6°, 40.9°, 49.5°, 54.1°, 62.4°, and 64.0° are attributed to the (012), (104), (110), (113), (024), (116), (214), and (300) planes of Fe_2_O_3_ (JCPDS, No. 33-0664), respectively. The XRD patterns of FCP700, FCP800, and FCP900 display the Fe_3_O_4_ and Fe diffraction peaks, indicating the formation of P-Fe_3_O_4_/Fe@C hybrids. The main diffraction peaks at 30.1°, 35.1°, 43.1°, 56.9°, and 62.5° are attributed to the (220), (311), (400), (511), and (440) planes of Fe_3_O_4_ (JCPDS, No. 74-0748), respectively [13]. The diffraction peaks at 44.6° and 65.0° are ascribed to the (110) and (200) planes of the standard cubic Fe (JCPDS, No. 87-0721), respectively. However, no diffraction peaks of iron phosphide are found in the XRD patterns of FCP700, FCP800, and FCP900, demonstrating the absence of iron phosphide in P-Fe_3_O_4_/Fe@C hybrids.

With the increase of thermal treatment temperature, the diffraction peaks' intensity of Fe_3_O_4_ decreases, and then the Fe increases. The ratio of Fe to Fe_3_O_4_ in the interface of P-Fe_3_O_4_/Fe@C can be adjusted by changing the thermal treatment temperature. With the increase of thermal treatment temperature, the ratio of Fe to Fe_3_O_4_ in P-Fe_3_O_4_/Fe@C interface increases. Rich under-coordinated Fe atoms in the interface of P-Fe_3_O_4_/Fe@C have a tremendous influence on the electrochemical water-splitting reaction. According to the BOLS–NEP theory [23], rich under-coordinated Fe atoms can adjust the electronic structure of P-Fe_3_O_4_/Fe@C, leading to the core electron entrapment and 3d orbital lone electron polarization, which can increase the local charge density and raise the occupied valence states towards the Fermi level of P-Fe_3_O_4_/Fe@C [26]. The coupling effect of entrapment and polarization can accelerate the water adsorption and activation to release more H* in the electrochemical water-splitting reaction [27,28].

XPS was performed to determine the functional group and valence information of P-Fe_3_O_4_/Fe@C (FCP800). As shown in Figure S2, all elements (P, C, O and Fe) can be found in the XPS survey spectrum, relating to the presence of P-Fe_3_O_4_/Fe@C. The Fe 2p XPS spectrum (Figure 2b) has four peaks at 710.6, 718.7, 724.0, and 730.5 eV, which are attributed to the Fe 2p_3/2_, Fe 2p_3/2_ sat, Fe 2p_1/2_, and Fe 2p_1/2_ sat, respectively, demonstrating the existence of Fe_3_O_4_ [18,29]. The satellite peaks at 710.6 and 724.0 eV may correspond to either the γ-Fe_2_O_3_ or Fe_3_O_4_ phase, because the Fe 2p_3/2_ and Fe 2p_1/2_ peaks are close for both phases. The peak at 718.7 corresponds to the γ-Fe_2_O_3_. The peak at 730.5 eV corresponds to the binding energy of Fe^2+^. The peak at 706.6 eV corresponds to the Fe 2p_3/2_ peak of the metallic Fe [16,30]. In the C 1s XPS spectrum for Figure 2c, the peaks at 283.4 and 284.8 eV correspond to C-C and C-O, respectively [31,32,33,34,35]. The peaks at 128.4 and 129.2 eV are attributed to P-Fe in the P 2p spectrum (Figure 2d) [16]. The broad peak at 132.5 eV corresponds to the oxidation product of P [36]. Results indicate that the P is successfully doped into the Fe_3_O_4_/Fe@C, and the prepared sample is P-Fe_3_O_4_/Fe@C.

**Figure 3:**
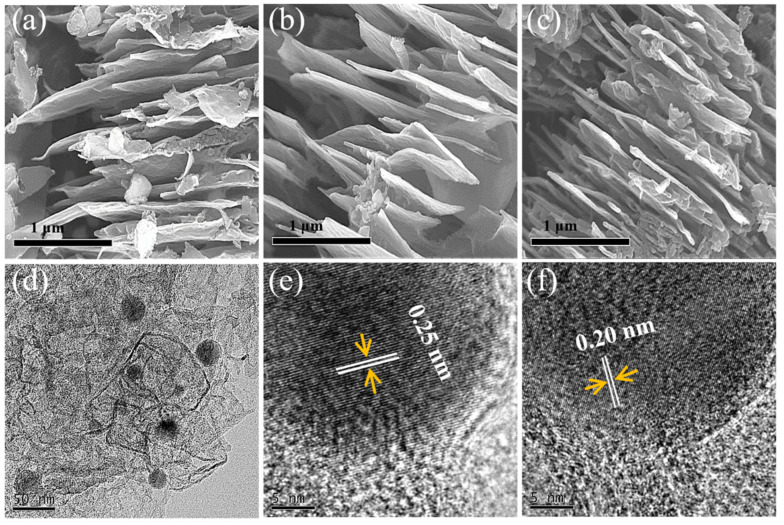
FESEM of (**a**) FCP700, (**b**) FCP800, and (**c**) FCP900; (**d**–**f**) HRTEM of FCP800.

Figure 3 shows the FESEM and HRTEM images of FCP700, FCP800, and FCP900. The morphology of FCP600 (not shown here) is similar to that of FCP700, FCP800, and FCP900. P-Fe_3_O_4_/Fe@C has nanolayered structures with the embedding of nanoparticles (Figure 3a–c). The size of nanoparticles and nanosheets are about 30 nm and 2 μm. FCP700, FCP800, and FCP900 display similar morphology, demonstrating that the thermal treatment temperature has no effect on the morphology of P-Fe_3_O_4_/Fe@C. The nanolayered structures with the embedding of nanoparticles are also found in the HRTEM of P-Fe_3_O_4_/Fe@C, which can provide high specific surface area and ion transport interface to improve the electrocatalytic activity. As shown in Figure 3d–f, the carbon support shows a layered structure, and the embedded nanoparticles are Fe and Fe_3_O_4_. The lattice fringe spacing in Figure 3e,f are 0.20 and 0.25 nm, corresponding to the (110) plane of Fe (JCPDS, No. 87-0721) and (311) plane of Fe_3_O_4_ (JCPDS, No. 74-0748), respectively.

**Figure 4:**
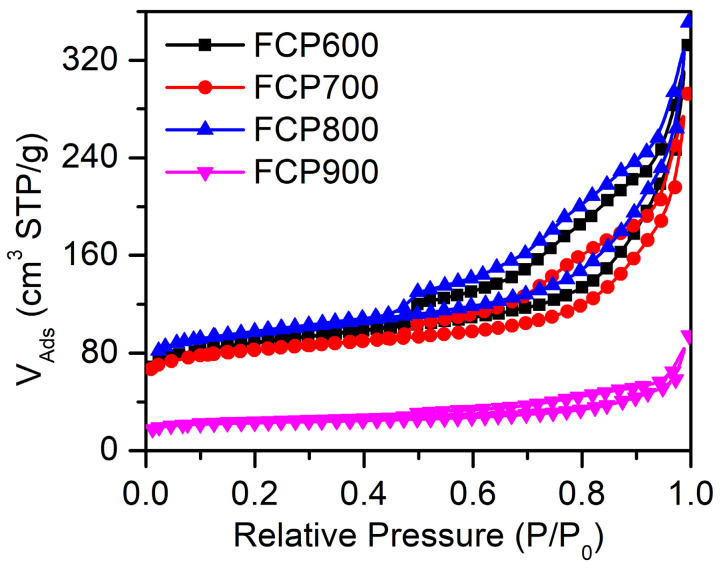
Nitrogen adsorption-desorption isotherms for FCP600, FCP700, FCP800, and FCP900.

The nitrogen adsorption–desorption isotherms of P-Fe_3_O_4_/Fe@C in Figure 4 display the Type IV curves with an H_3_ hysteresis loop, demonstrating the presence of mesoporous structures. The specific surface areas, pore volume, and average pore size of FCP600, FCP700, FCP800, and FCP900 are summarized in Table 1. The average pore diameters of FCP600, FCP700, FCP800, and FCP900 have no obvious effect on the electrocatalytic reaction process. The porosity plays a crucial role in electrocatalysis by enhancing the accessibility of reactants to active sites, facilitating the mass transport of reactants and products, increasing the specific surface area to provide more active sites, and improving charge transfer. Therefore, the largest specific surface area and mesoporous structure for FCP800 can provide a transport channel and more reaction-active sites, thus lowering the overpotential at a low current density, which represents the conditions of a relatively small driving force [37].

However, when the current density increases to the visible industry-relevant levels, the relationship between porosity and overpotential can change. The mass transport limitation becomes more prominent as the current density increases. At the visible industry-relevant current density, deep pores are blocked or inaccessible due to the slow mass transport inside, which can result in reactant depletion near the catalyst surface and hinder the efficient access of reactants to the active sites, thus increasing the overpotential [38]. Therefore, the general effect of porosity on a certain activity of electrocatalyst is very complex and non-linear. The reaction conditions, such as the nature of the reactants, electrolyte composition, temperature, and current density, are crucial for interpreting the relationship between porosity and electrochemical performance accurately.

### 3.2. Electrocatalytic Activity of HER

**Figure 5:**
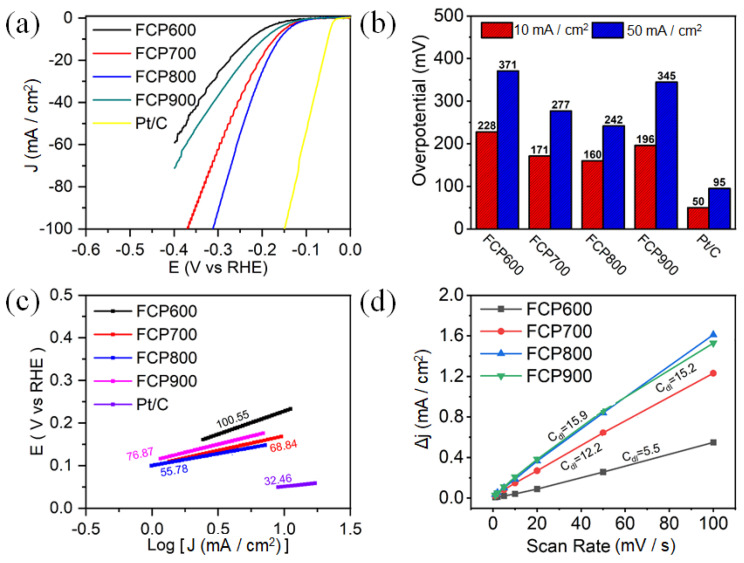
HER electrocatalytic activity in 0.5 M H_2_SO_4_ electrolyte: (**a**) LSV curves, (**b**) corresponding η, (**c**) Tafel plots, and (**d**) C_dl_ of Pt/C and P-Fe_3_O_4_/Fe@C.

The electrocatalytic activity of P-Fe_3_O_4_/Fe@C was evaluated in 1.0 M KOH and 0.5 M H_2_SO_4_ electrolytes using a standard three-electrode system. The HER activities of Pt/C and the Fe_3_O_4_/Fe@C electrocatalyst were also tested for comparison, as shown in Figure S3. The Fe_3_O_4_/Fe@C shows a low overpotential with 218 and 280 mV and Tafel slope with 65.5 and 176.26 mV dec^−1^ in the acidic and alkaline electrolytes. Figure 5a shows the linear sweep voltammetry (LSV) curves of Pt/C and P-Fe_3_O_4_/Fe@C in the 0.5 M H_2_SO_4_ electrolyte. P-Fe_3_O_4_/Fe@C shows good HER electrocatalytic activity. The thermal treatment temperature has an impact on the HER performance of P-Fe_3_O_4_/Fe@C. With the increase of thermal treatment temperature, the HER performance of P-Fe_3_O_4_/Fe@C increases and then decreases. For Figure 5b, FCP800 shows the best HER activity with the lowest overpotential (η) of 160 mV at 10 mA cm^−2^, compared to those of FCP600 (228 mV), FCP700 (171 mV), and FCP900 (196 mV). It is worth mentioning that P-Fe_3_O_4_/Fe@C (FCP800) shows outstanding electrocatalytic activity, even under the high current density of 50 mA cm^−2^ at overpotentials of 242 mV in the acidic medium. The HER-specific activity of FCP600, FCP700, FCP800, and FCP900 are 2.50, 5.13, 7.56, and 3.92 mA cm^−2^ at the overpotential of 150 mV in 0.5 M H_2_SO_4_ electrolyte, respectively (Figure S4a). The HER mass activity of FCP600, FCP700, FCP800, and FCP900 are 2.81, 4.29, 4.47, and 2.08 A mg^−1^ at the overpotential of 150 mV in 0.5 M H_2_SO_4_ electrolyte, respectively (Figure S4b). The electrocatalytic reaction kinetics of P-Fe_3_O_4_/Fe@C was studied by means of the Tafel plots constructed from the LSV curves (Figure 5c). The Tafel slope values for FCP600, FCP700, FCP800, and FCP900 are 100.55, 68.84, 55.78, and 76.87 mV dec^−1^, respectively. The relatively smaller Tafel slope for FCP800 demonstrates the fast HER reaction kinetics. The double-layer capacitance (C_dl_) was evaluated by the CV curves with different scan rates (Figures S5 and S6). For Figure 5d, the C_dl_ values for FCP600, FCP700, FCP800, and FCP900 are 5.5, 12.2, 15.9, and 15.2 mF cm^−2^, respectively. The Tafel plots and C_dl_ results are consistent with the electrocatalytic activity. The electrochemical active surface area (ECSA) values of FCP600, FCP700, FCP800, and FCP900 are 137.5, 305.0, 397.5, and 380.0 in the acidic electrolyte, respectively.

**Figure 6:**
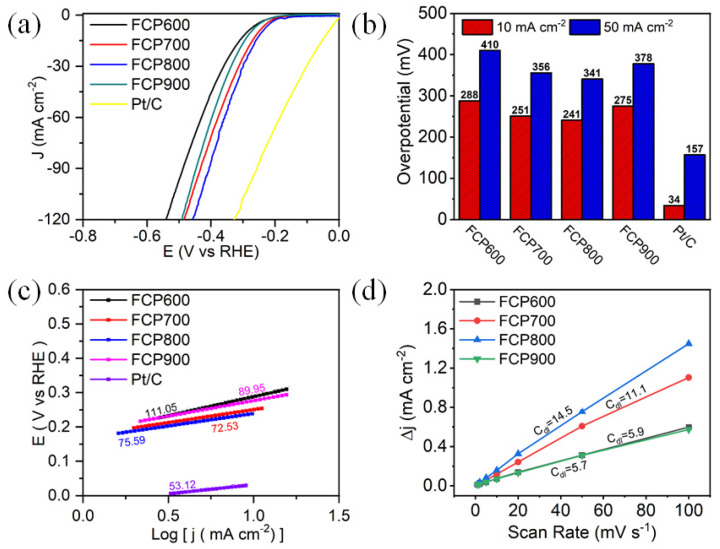
HER electrocatalytic activity in 1.0 M KOH electrolyte: (**a**) LSV curves, (**b**) corresponding η, (**c**) Tafel plots, and (**d**) C_dl_ of Pt/C and P-Fe_3_O_4_/Fe@C.

**Figure 7:**
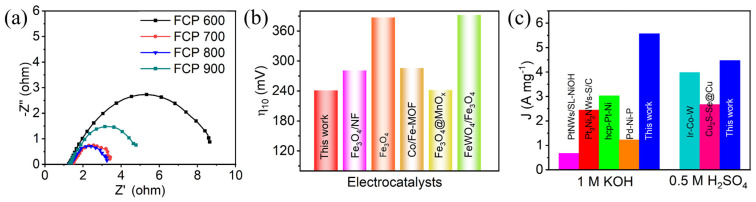
(**a**) Nyquist plots of FCP600, FCP700, FCP800, and FCP900 electrodes; comparison of (**b**) electrocatalytic activity of Fe-based electrocatalysts in 1.0 M KOH electrolyte and (**c**) mass activity of HER electrocatalysts in 1.0 M KOH electrolyte.

The electrocatalytic activity of P-Fe_3_O_4_/Fe@C was also studied in 1.0 M KOH electrolyte using a standard three-electrode system (Figure 6). Similar electrocatalytic activity results in Figure 6a can be found in alkaline media. As shown in Figure 6b, FCP800 shows the lowest η of 214 mV at 10 mA cm^−2^, compared to those of FCP600 (288 mV), FCP700 (251 mV), and FCP900 (275 mV). Notably, P-Fe_3_O_4_/Fe@C (FCP800) achieves a current density of 50 mA cm^−2^ at an overpotential of 341 mV in the alkaline medium. The HER-specific activity of FCP600, FCP700, FCP800, and FCP900 are 0.08, 3.56, 7.89, and 2.34 mA cm^−2^ at the overpotential of 150 mV in 1.0 M KOH electrolyte, respectively (Figure S4a). The HER mass activity of FCP600, FCP700, FCP800, and FCP900 are 1.62, 2.4, 5.57, and 0.13 A mg^−1^ at the overpotential of 150 mV in 1.0 M KOH electrolyte, respectively (Figure S4b). The Tafel slope value of FCP800 is 75.59 mV dec^−1^, which is much smaller than those of FCP600 (111.05 mV dec^−1^) and FCP900 (89.95 mV dec^−1^), indicating its superior HER reaction kinetics (Figure 6c). As shown in Figure 6d, FCP800 exhibits the highest C_dl_ of 14.5 mF cm^−2^, followed by FCP600 (5.7 mF cm^−2^), FCP700 (11.1 mF cm^−2^), and FCP900 (5.9 mF cm^−2^). The electrochemical active surface area (ECSA) values of FCP600, FCP700, FCP800, and FCP900 are 142.5, 147.5, 362.5, and 277.5 in an alkaline electrolyte, respectively. Electrochemical impedance spectroscopy (EIS) was used to investigate the electron transfer mechanism. Figure 7a displays the Nyquist plots of FCP600, FCP700, FCP800, and FCP900 electrodes. The values of R_s_, R_ct_, and CPE are fitted and listed in Table S1. The small semicircle diameter in the high frequency region of the Nyquist plot indicates a low charge transfer resistance (R_ct_) at the electrode–electrolyte interface, which suggests a faster charge transfer kinetics and reaction rate. With the increase of Fe content in P-Fe_3_O_4_/Fe@C interface, the R_ct_ decreases and then increases. The R_ct_ value of the FCP800 electrodes is lowest, demonstrating a favorable charge transfer due to their excellent electronic conductivity. Therefore, P-Fe_3_O_4_/Fe@C shows excellent electrocatalytic HER activity and mass activity compared to previously reported Fe-based electrocatalysts in the literature, as shown in Figure 7b,c.

**Figure 8:**
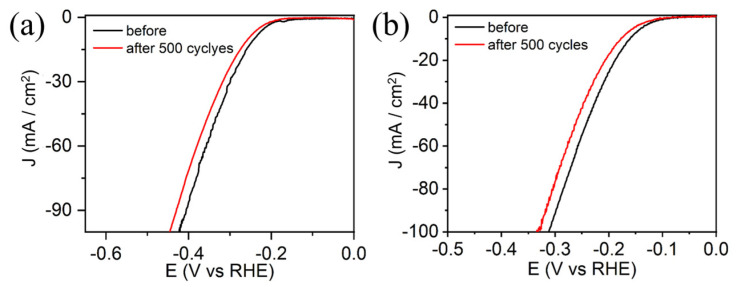
LSV curves before and after 500 cycles of P-Fe_3_O_4_/Fe@C (FCP800) in (**a**) 1.0 M KOH and (**b**) 0.5 M H_2_SO_4_ electrolytes.

The electrocatalytic stability of electrocatalysts is very important in practical application. In particular, MOF materials are prone to destruction under acidic media. The electrocatalytic stability of P-Fe_3_O_4_/Fe@C (FCP800) was studied by a CV test. The corresponding LSV curves both before and after 500 cycles are shown in Figure 8. It can be unambiguously found that the LSV curves of FCP800 before and after 500 cycles vary very little in acidic and alkaline electrolytes, demonstrating the good electrocatalytic stability of P-Fe_3_O_4_/Fe@C. The XRD patterns of P-Fe_3_O_4_/Fe@C (FCP800) after 500 cycles in 1.0 M KOH and 0.5 M H_2_SO_4_ electrolytes have been provided, as shown in Figure S7. Results indicate that P-Fe_3_O_4_/Fe@C shows good structural stability and durability under the tested conditions, making it a promising material for various electrochemical applications.

FCP800 shows superior electrocatalytic activity with a relatively low overpotential and Tafel slope as well as remarkable stability due to the rich under-coordinated Fe atoms in the P-Fe_3_O_4_/Fe@C interfaces. The reasons for the enhancement of electrocatalytic activity for FCP800 are as follows: (i) rich under-coordinated Fe atoms can result in core electron entrapment and 3d orbital lone electron polarization, which can increase the local charge density and raise the occupied valence states towards the Fermi level of P-Fe_3_O_4_/Fe@C. The coupling effect of entrapment and polarization can promote the water adsorption and activation to release more H* in the electrochemical water-splitting reaction; (ii) an appropriate under-coordinated Fe atom proportion in the Fe_3_O_4_/Fe@C interfaces can increase the specific surface area and pore volume, which can provide more reaction active sites and transport channels for water molecular adsorption and electron/ion transfer, thus lowering the overpotential to a low current density; (iii) an appropriate under-coordinated Fe atom proportion can improve the electronic conductivity and reduce the charge transfer impedance of Fe_3_O_4_/Fe@C, which facilitate efficient electron transfer, allowing for faster electrocatalytic reaction kinetics. Meanwhile, the carbon support can also improve the electron transfer efficiency in the electrocatalytic reaction process [39,40].

## 4. Conclusions

In summary, P-Fe_3_O_4_/Fe@C has been synthesized by an hydrothermal method and pyrolysis for pH-universal water splitting. The proper under-coordinated Fe atoms in the P-Fe_3_O_4_/Fe@C interfaces can effectively enhance the electrocatalytic HER activity. Rich under-coordinated Fe atoms lead to an increased local charge density, core electron entrapment, and lone electron polarization, which can accelerate water adsorption and release more H* in the electrochemical water-splitting reaction. As a donor-like catalyst, P-Fe_3_O_4_/Fe@C has low overpotentials of 160 mV in acidic media and 214 mV in alkaline media at 10 mA cm^−2^ and shows an excellent pH-universal electrocatalytic stability.

## Figures and Tables

**Table 1:** Specific surface areas, pore volumes, and average pore diameters for FCP600, FCP700, FCP800, and FCP900.

Sample	Specific Surface Areas (m^2^ g^−1^)	Pore Volume (cm^3^ g^−1^)	Average Pore Diameters (nm)
FCP600	322.073	0.5141	3.911
FCP700	302.940	0.4521	3.915
FCP800	363.318	0.5427	3.913
FCP900	87.306	0.146	3.911

## Data Availability

Data will be made available on request.

## Supplementary Materials

The following supporting information can be downloaded at: https://www.mdpi.com/article/10.3390/nano13131909/s1. Figure S1: XRD pattern of P-Fe_3_O_4_/Fe@C (FCP600). Figure S2: XPS survey spectrum of P-Fe_3_O_4_/Fe@C (FCP800). Figure S3: HER electrocatalytic activity in (a,b) 0.5 M H_2_SO_4_ and (c,d) 1.0 M KOH electrolytes: (a–c) LSV curves and (b–d) Tafel plots of Fe_3_O_4_/Fe@C and FCP800. Figure S4: (a) HER-specific activity and (b) mass activity of FCP600, FCP700, FCP800, and FCP900 in the 0.5 M H_2_SO_4_ and KOH electrolytes. Figure S5: CV curves of (a) FCP600, (b) FCP700, (c) FC800, and (d) FCP900 electrodes at different scan rates in 0.5 M H_2_SO_4_ solution. Figure S6: CV curves of (a) FCP600, (b) FCP700, (c) FC800, and (d) FCP900 electrodes at different scan rates in 1.0 M KOH solution. Figure S7: XRD patterns of P-Fe_3_O_4_/Fe@C (FCP800) after 500 cycles in 1.0 M KOH and 0.5 M H_2_SO_4_ electrolytes. Table S1: The fitted R_s_ and R_ct_ for FCP600, FCP700, FCP800, and FCP900.

## References

[1] Wu F., Guo X.X., Wang Q.H., Lu S.W., Wang J.L., Hu Y.B., Hao G.Z., Li Q.L., Yang M.Q., Jiang W. (2020). A hybrid of MIL-53(Fe) and conductive sulfide as a synergistic electrocatalyst for the oxygen evolution reaction. J. Mater. Chem. A.

[2] Fu C.Y., Hao W.J., Fan J.L., Zhang Q., Guo Y.H., Fan J.C., Chen Z.L., Li G.S. (2023). Fabrication of Ultra-Durable and Flexible NiP_x_-Based Electrode toward High-Efficient Alkaline Seawater Splitting at Industrial Grade Current Density. Small.

[3] Shen S.L., Gao P.J., Chen H., Tang Z.H., Li J., Xiu H.X., Yang J.H. (2023). Graphited carbon black curled nanoribbons simultaneously boosted stability and electrocatalytic activity of 1T-MoS_2_/MoO_3_ toward hydrogen evolution. J. Alloys Compd..

[4] Kim M.G., Choi Y.H. (2023). Electrocatalytic Properties of Co_3_O_4_ Prepared on Carbon Fibers by Thermal Metal-Organic Deposition for the Oxygen Evolution Reaction in Alkaline Water Electrolysis. Nanomaterials.

[5] Salvo D., Mosconi D., Neyman A., Bar-Sadan M., Calvillo L., Granozzi G., Cattelan M., Agnoli S. (2023). Nanoneedles of Mixed Transition Metal Phosphides as Bifunctional Catalysts for Electrocatalytic Water Splitting in Alkaline Media. Nanomaterials.

[6] Liang R.K., Fan J.L., Lei F.J., Li P., Fu C.Y., Lu Z.K., Hao W.J. (2023). Fabrication of ultra-stable and high-efficient CoP-based electrode toward seawater splitting at industrial-grade current density. J. Colloid Interface Sci..

[7] Cebollada J., Sebastian D., Lazaro M.J., Martinez-Huerta M.V. (2023). Carbonized Polydopamine-Based Nanocomposites: The Effect of Transition Metals on the Oxygen Electrocatalytic Activity. Nanomaterials.

[8] Zeng X.S., Tu Z.X., Yuan Y.L., Liao L.L., Xiao C.C., Wen Y.F., Xiong K. (2022). Two-Dimensional Transition Metal-Hexaaminobenzene Monolayer Single-Atom Catalyst for Electrocatalytic Carbon Dioxide Reduction. Nanomaterials.

[9] Zhang X.J., Ou-Yang W., Zhu G., Lu T., Pan L.K. (2019). Shuttle-like carbon-coated FeP derived from metal-organic frameworks for lithium-ion batteries with superior rate capability and long-life cycling performance. Carbon.

[10] Yang M., Hu W.H., Li M.X., Cao Y.N., Dong B., Ma Y., Zhao H.Y., Wang F.G., Huang J.E., Chai Y.M. (2022). Controlled high-density interface engineering of Fe_3_O_4_-FeS nanoarray for efficient hydrogen evolution. J. Energy Chem..

[11] Liu Y.F., Li Y., Wu Q., Su Z., Wang B., Chen Y.F., Wang S.F. (2021). Hollow CoP/FeP_4_ Heterostructural Nanorods Interwoven by CNT as a Highly Efficient Electrocatalyst for Oxygen Evolution Reactions. Nanomaterials.

[12] Ma F.X., Xu C.Y., Lyu F.C., Song B., Sun S.C., Li Y.Y., Lu J., Zhen L. (2019). Construction of FeP Hollow Nanoparticles Densely Encapsulated in Carbon Nanosheet Frameworks for Efficient and Durable Electrocatalytic Hydrogen Production. Adv. Sci..

[13] Pan X., Wang W.J., Chen Y., Wen Q., Li X.Q., Lin C.H., Wang J.H., Xu H.T., Yang L.Q.Y. (2022). Bio-electrocatalyst Fe_3_O_4_/Fe@C derived from MOF as a high-performance bioanode in single-chamber microbial fuel cell. Biochem. Eng. J..

[14] Ramachandra S.K., Nagaraju D.H., Marappa S., Kapse S., Thapa R. (2022). Highly efficient catalysts of ruthenium clusters on Fe_3_O_4_/MWCNTs for the hydrogen evolution reaction. New J. Chem..

[15] Saleh M.R., Thabet S.M., El-Gendy R.A., Saleh M., El-Bery H.M. (2022). MIL-53 (Fe) for constructing hydrogenated Fe_3_O_4_@C@TiO_2_ double core-shell nanocrystals as superior bifunctional photocatalyst. J. Photochem. Photobiol. A Chem..

[16] Zhang X.Y., Li F.T., Fan R.Y., Zhao J., Dong B., Wang F.L., Liu H.J., Yu J.F., Liu C.G., Chai Y.M. (2021). F, P double-doped Fe_3_O_4_ with abundant defect sites for efficient hydrogen evolution at high current density. J. Mater. Chem. A.

[17] Zhang J.Q., Shang X., Ren H., Chi J.Q., Fu H., Dong B., Liu C.G., Chai Y.M. (2019). Modulation of Inverse Spinel Fe_3_O_4_ by Phosphorus Doping as an Industrially Promising Electrocatalyst for Hydrogen Evolution. Adv. Mater..

[18] Xu W.L., Zhong W.D., Yang C.F., Zhao R., Wu J., Li X.K., Yang N.J. (2022). Tailoring interfacial electron redistribution of Ni/Fe_3_O_4_ electrocatalysts for superior overall water splitting. J. Energy Chem..

[19] Adamson W., Bo X., Li Y.B., Suryanto B.H.R., Chen X.J., Zhao C. (2020). Co-Fe binary metal oxide electrocatalyst with synergistic interface structures for efficient overall water splitting. Catal. Today.

[20] Srinivas K., Lu Y.J., Chen Y.F., Zhang W.L., Yang D.X. (2020). FeNi_3_-Fe_3_O_4_ Heterogeneous Nanoparticles Anchored on 2D MOF Nanosheets/1D CNT Matrix as Highly Efficient Bifunctional Electrocatalysts for Water Splitting. ACS Sustain. Chem. Eng..

[21] Mirabella F., Müllner M., Touzalin T., Riva M., Jakub Z., Kraushofer F., Schmid M., Koper M.T.M., Parkinson G.S., Diebold U. (2021). Ni-modified Fe_3_O_4_ (001) surface as a simple model system for understanding the oxygen evolution reaction. Electrochim. Acta.

[22] Meng S.C., Sun S.C., Liu Y., Lu Y.K., Chen M. (2022). Synergistic modulation of inverse spinel Fe_3_O_4_ by doping with chromium and nitrogen for efficient electrocatalytic water splitting. J. Colloid Interface Sci..

[23] Liu X.J., Zhang X., Bo M.L., Li L., Tian H.W., Nie Y.G., Sun Y., Xu S.Q., Wang Y., Zheng W.T. (2015). Coordination-Resolved Electron Spectrometrics. Chem. Rev..

[24] Hou S.J., Xu X.T., Wang M., Lu T., Sun C.Q., Pan L.K. (2018). Synergistic conversion and removal of total Cr from aqueous solution by photocatalysis and capacitive deionization. Chem. Eng. J..

[25] Qiang T.T., Chen L., Qin X.T. (2021). Biomass-based 0D/3D N-CQD/MIL-53(Fe) photocatalyst for the simultaneous remediation of multiple hazardous pollutants in sewage. Catal. Sci. Technol..

[26] Sun C.Q. (2020). The BOLS-NEP theory reconciling the attributes of undercoordinated adatoms, defects, surfaces and nanostructures. Nano Mater. Sci..

[27] Sun C.Q. (2021). Perspective: Bonding and electronic origin of Au atomic-undercoordination-derivacy and nanoscale-size-dependency. Vacuum.

[28] Peng W.W., Pan X.R., Liu X.J., Gao Y., Lu T., Li J.B., Xu M., Pan L.K. (2023). A moisture self-regenerative, ultra-low temperature anti-freezing and self-adhesive polyvinyl alcohol/polyacrylamide/CaCl_2_/MXene ionotronics hydrogel for bionic skin strain sensor. J. Colloid Interface Sci..

[29] Xu L.M., Ding Z.B., Chen Y.Y., Xu X.T., Liu Y., Li J.B., Lu T., Pan L.K. (2023). Carbon nanotube bridged nickel hexacyanoferrate architecture for high-performance hybrid capacitive deionization. J. Colloid Interface Sci..

[30] Zhang S.H., Wu M.F., Tang T.T., Xing Q.J., Peng C.Q., Li F., Liu H., Luo X.B., Zou J.P., Min X.B. (2018). Mechanism investigation of anoxic Cr(VI) removal by nano zero-valent iron based on XPS analysis in time scale. Chem. Eng. J..

[31] Liang R.W., Jing F.F., Shen L.J., Qin N., Wu L. (2015). MIL-53(Fe) as a highly efficient bifunctional photocatalyst for the simultaneous reduction of Cr(VI) and oxidation of dyes. J. Hazard. Mater..

[32] Meng F.Y., Ding Z.B., Chen Z.Q., Wang K., Liu X.J., Li J.F., Lu T., Xu X.T., Pan L.K. (2022). N-doped carbon@Cu core-shell nanostructure with nearly full solar spectrum absorption and enhanced solar evaporation efficiency. J. Mater. Chem. A.

[33] Wang K., Liu Y., Xu X.T., Jiao Y., Pan L.K. (2023). In situ synthesis of ultrasmall NaTi_2_(PO_4_)_3_ nanocube decorated carbon nanofiber network enables ultrafast and superstable rocking-chair capacitive deionization. Chem. Eng. J..

[34] Liu D., Li C.L., Zhao C.Y., Zhao Q., Niu T.Q., Pan L.K., Xu P.W., Zhang F.Q., Wu W.D., Ni T.J. (2022). Facile synthesis of three-dimensional hollow porous carbon doped polymeric carbon nitride with highly efficient photocatalytic performance. Chem. Eng. J..

[35] Liu X.H., Xu X.T., Xuan X.X., Xia W., Feng G.L., Zhang S.H., Wu Z.G., Zhong B.H., Guo X.D., Xie K.Y. (2023). Unlocking Enhanced Capacitive Deionization of NaTi_2_(PO_4_)_3_/Carbon Materials by the Yolk-Shell Design. J. Am. Chem. Soc..

[36] Ding Z.B., Yu H.Z., Liu X.J., He N.N., Chen X.H., Li H.B., Wang M., Yamauchi Y., Xu X.T., Amin M.A. (2022). Prussian blue analogue derived cobalt-nickel phosphide/carbon nanotube composite as electrocatalyst for efficient and stable hydrogen evolution reaction in wide-pH environment. J. Colloid Interface Sci..

[37] Qi J., Zhang W., Cao R. (2018). Porous Materials as Highly Efficient Electrocatalysts for the Oxygen Evolution Reaction. ChemCatChem.

[38] Jin M.T., Zhang X., Niu S.Z., Wang Q., Huang R.Q., Ling R.H., Huang J.Q., Shi R., Amini A., Cheng C. (2022). Strategies for Designing High-Performance Hydrogen Evolution Reaction Electrocatalysts at Large Current Densities above 1000 mA cm^–2^. ACS Nano..

[39] Bertran-Serra E., Musheghyan-Avetisyan A., Chaitoglou S., Amade-Rovira R., Alshaikh I., Pantoja-Suarez F., Andujar-Bella J., Jawhari T., Perez-del-Pino A., Gyorgy E. (2023). Temperature-modulated synthesis of vertically oriented atomic bilayer graphene nanowalls grown on stainless steel by inductively coupled plasma chemical vapour deposition. Appl. Surf. Sci..

[40] Chaitoglou S., Amade R., Bertran E. (2022). Insights into the inherent properties of vertical graphene flakes towards hydrogen evolution reaction. Appl. Surf. Sci..

