# Supplementary material for: Interface Coordination Engineering of P-Fe3O4/Fe@C Derived from an Iron-Based Metal Organic Framework for pH-Universal Water Splitting
Source: Nanomaterials (Basel). 2023 Jun 22;13(13):1909. doi: 10.3390/nano13131909 (PMC10343528; doi:10.3390/nano13131909)
Supplement: Supplementary file 1 [file nanomaterials-13-01909-s001.zip › nanomaterials-2429257-supplementary.pdf]

nanomaterials

MDPI

Supplementary Materials

Interface Coordination Engineering of P-Fe_3O_4/Fe@C Derived from an Iron-Based Metal Organic Framework for pH-Universal Water Splitting

Minmin Fan [1], Peixiao Li [2], Baibai Liu [3], Yun Gong [1], Chengling Luo [1], Kun Yang [1], Xinjuan Liu [1,*], Jinchen Fan [1] and Yuhua Xue [1,*]

[1] School of Materials and Chemistry, University of Shanghai for Science and Technology, Shanghai 200093, China; fanminmin1998@163.com (M.F.); 211590181@st.usst.edu.cn (Y.G.); clingluo@163.com (C.L.); yangkun199800@163.com (K.Y.); xueyuhua@usst.edu.cn (Y.X.)
[2] Beijing Smartchip Microelectronics Technology Company Limited, Beijing 102200, China; pricheer@126.com
[3] Key Laboratory of Optoelectronic Technology & Systems (Ministry of Education), College of Optoelectronic Engineering, Chongqing University, Chongqing 400044, China; 15067132581@163.com
* Correspondence: lxj669635@126.com (X.L.)

Characterization

The phase and crystal structure of samples was characterized by X-ray diffraction (XRD, Rigaku Ultima IV) with Cu Kα radiation (30 kV, 25 mA). The surface chemical state and composition of samples was performed by X-ray photoelectron spectroscopy (XPS) using a Thermo ESCALAB 250Xi spectrometer with a monochromatic Al Kα X-ray source. The morphology of samples was characterized by field-emission scanning electron microscopy (FESEM, Quanta FEG 450) and transmission electron microscopy (HRTEM, FEI TF20). The nitrogen adsorption isotherms were measured using a BELSORP-max nitrogen adsorption apparatus (Micrometitics, Norcross, GA) at 77 K.

Electrochemical measurements

The electrochemical activity of as-prepared samples was evaluated on the electrochemical workstation (CHI 760E) in the three-electrode system. The working electrodes was obtained as following. The as-prepared catalysts (10 mg) was dispersed in Nafion 117 solution (5 wt%) under magnetic stirring for 20 min to obtain a uniform dispersion. Then, the suspension was coated on the glassy carbon electrode. The prepared electrodes with mass loading of 0.2 mg cm^{-2} were dried at the room temperature. The carbon rod with diameter of 6 mm was used as counter electrode. Hg/HgO electrode and standard calomel electrode (SCE) were used as reference electrodes for alkaline and acidic media, respectively. 1.0 M KOH and 0.5 M H_2SO_4 aqueous solution (50 mL) were used as alkaline and acidic electrolytes, respectively. In order to assess the potential *versus* reversible hydrogen electrode (RHE), the potential *versus* reference electrode was calibrated by the following equation: $E_{RHE} = E_{RE} + 0.059*pH + 0.245$ (SCE)/0.098 (Hg/HgO). Linear sweep voltammetry (LSV) was measured at a scanning rate of 2 mV s^{-1}. Tafel slopes are calculated from the LSV polarization curves. The electrochemical impedance spectroscopy (EIS) was examined in the frequency region from 10^5 to 10^{-2} Hz with the amplitude of 5 mV. The double-layer capacitance (C_{dl}) is obtained by a series of cyclic voltammetry (CV) tests at different scanning rates in the non-faradic region. The electrochemical active surface area (ECSA) of an electrode is calculated using the following equation:

$$\mathrm{ECSA} = \frac{C_{dl}}{40\ \mu F\ cm^{-2}}$$

The electrocatalytic stability was evaluated by CV test in alkaline and acidic solutions.

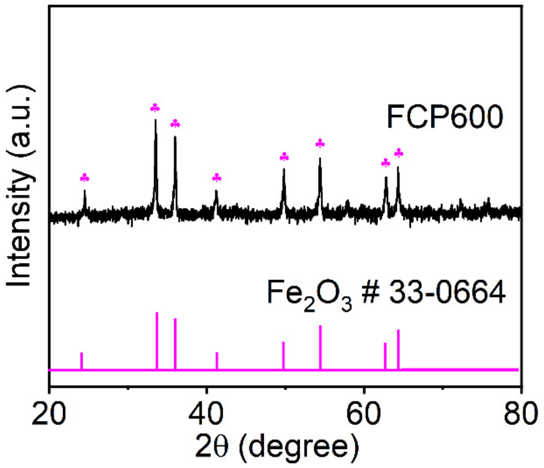

Figure S1. XRD pattern of P-Fe_3O_4/Fe@C (FCP600).

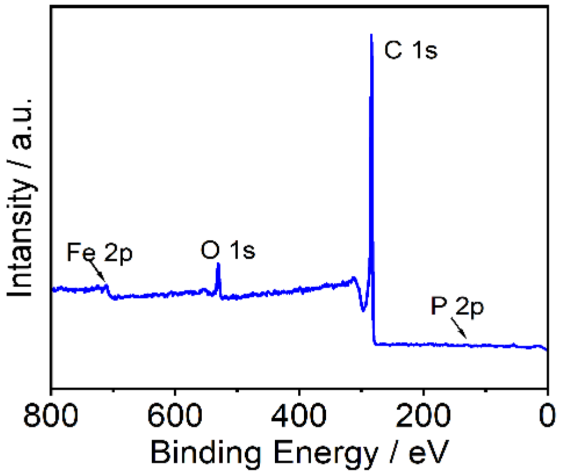

Figure S2. XPS survey spectrum of P-Fe_3O_4/Fe@C (FCP800).

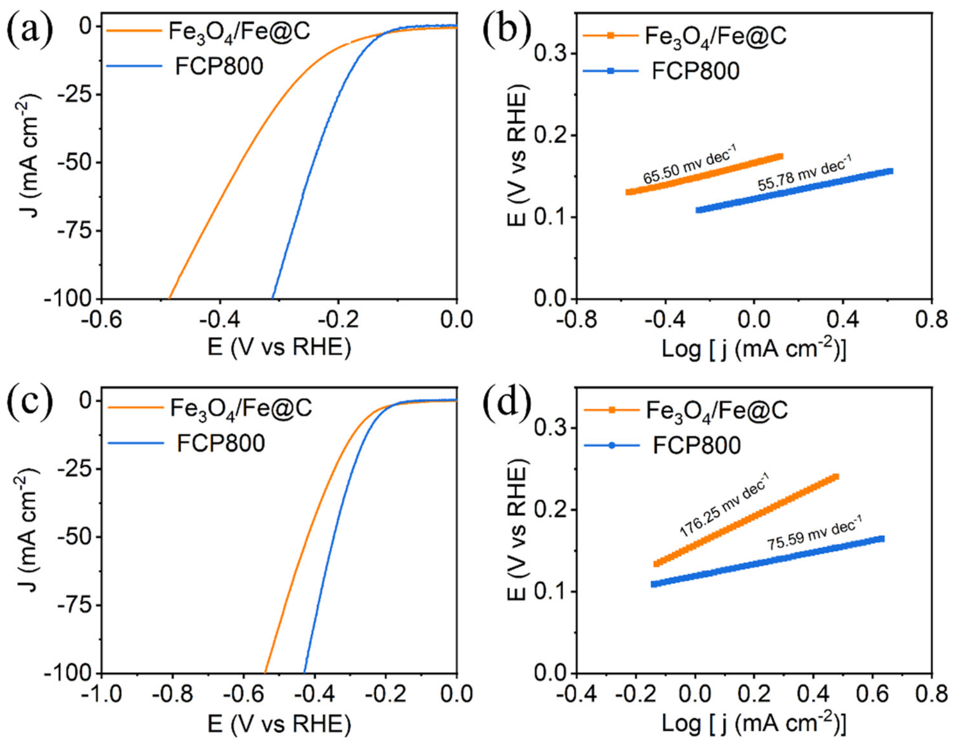

Figure S3. HER electrocatalytic activity in (**a**,**b**) 0.5 M H_2SO_4 and (**c**,**d**) 1.0 M KOH electrolytes: (**a**-**c**) LSV curves and (**b**-**d**) Tafel plots of Fe_3O_4/Fe@C and FCP800.

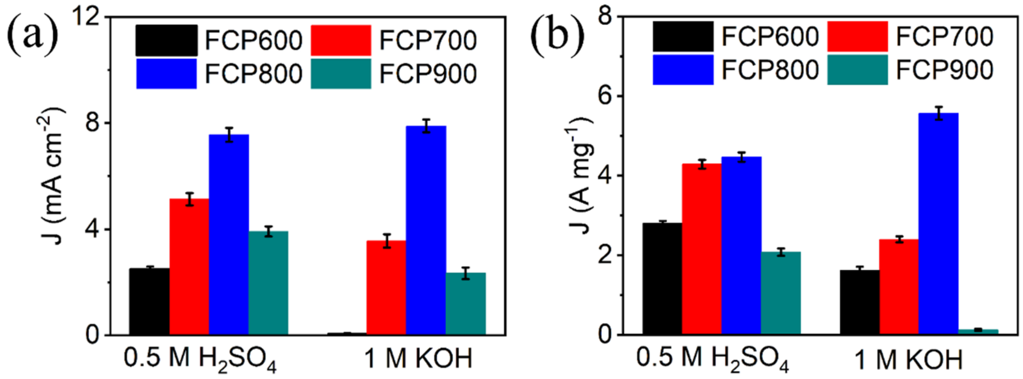

Figure S4. (**a**) HER specific activity and (**b**) mass activity of FCP600, FCP700, FCP800 and FCP900 in the 0.5 M H_2SO_4 and KOH electrolytes.

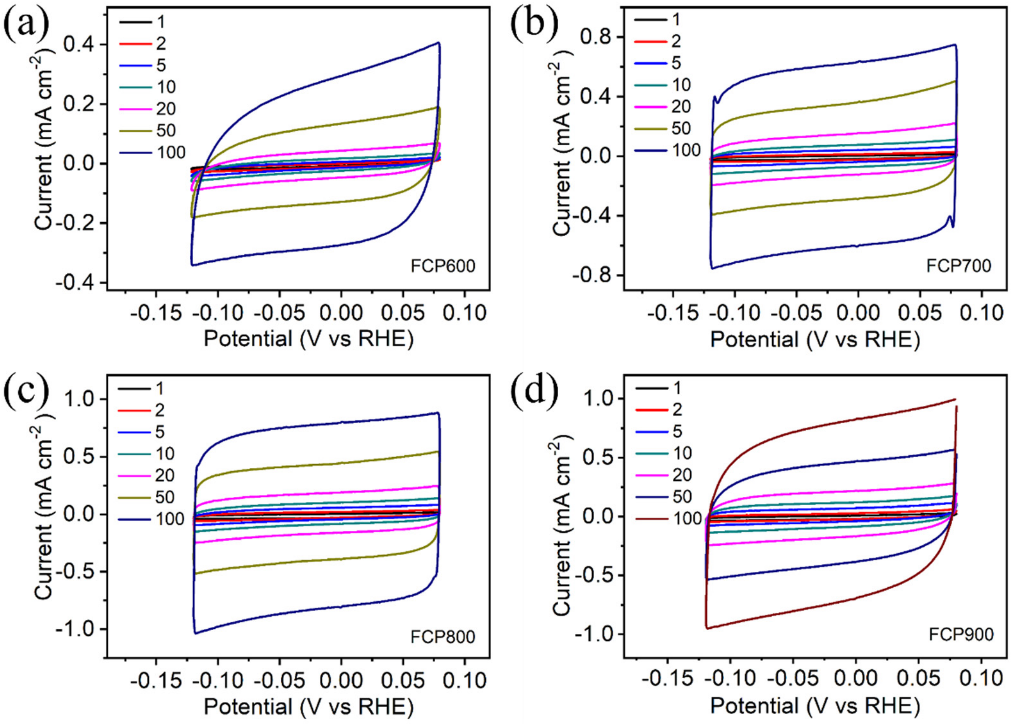

Figure S5. CV curves of (**a**) FCP600, (**b**) FCP700, (**c**) FC800, and (**d**) FCP900 electrodes at different scan rates in 0.5 M H_2SO_4 solution.

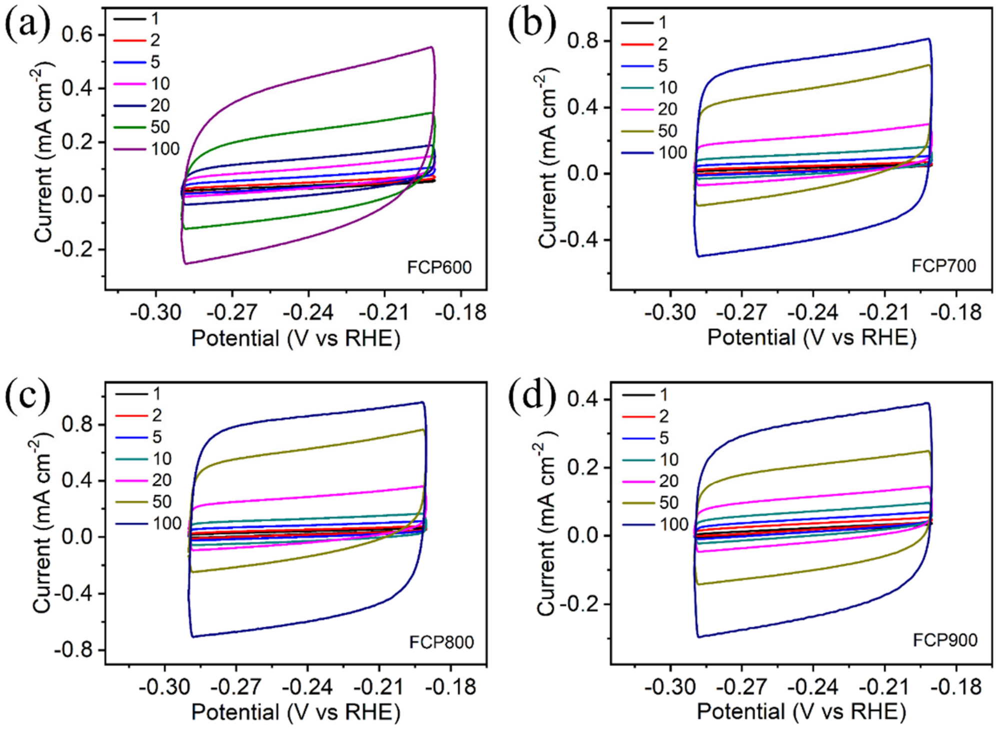

Figure S6. CV curves of (**a**) FCP600, (**b**) FCP700, (**c**) FC800, and (**d**) FCP900 electrodes at different scan rates in 1.0 M KOH solution.

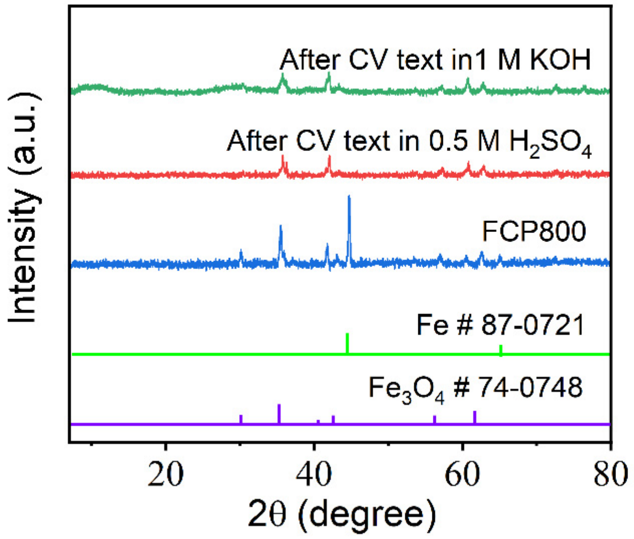

Figure S7. XRD patterns of P-Fe_3O_4/Fe@C (FCP800) after 500 cycles in 1.0 M KOH and 0.5 M H_2SO_4 electrolytes.

Table S1. The fitted R_s and R_{ct} for FCP600, FCP700, FCP800 and FCP900.

Sample	R_s(Ω)	R_{ct}(Ω)
FCP600	1.67	7.52
FCP700	1.46	2.17
FCP800	1.24	1.58
FCP900	1.12	3.69
